# Circular RNA hsa_circ_0032463 Acts as the Tumor Promoter in Osteosarcoma by Regulating the MicroRNA 498/LEF1 Axis

**DOI:** 10.1128/MCB.00100-21

**Published:** 2021-07-23

**Authors:** Guanghua Qin, Xuejian Wu

**Affiliations:** a Department of Orthopedics, The First Affiliated Hospital of Zhengzhou University, Zhengzhou, Henan, China

**Keywords:** circ_0032463, miR-498, LEF1, osteosarcoma, proliferation, apoptosis, migration

## Abstract

Several studies have examined the relationship between osteosarcoma (OS) and microRNAs (miRNAs). However, only a few researchers have investigated the underlying mechanism of circular RNAs (circRNAs) in OS development. Our paper aimed to assess how hsa_circ_0032463 (abbreviated “circ_0032463” here) initiates and regulates OS progression. We detected circ_0032463 expression in OS tissues and cell lines by using reverse transcription-quantitative PCR (RT-qPCR) analysis and then investigated the interaction between circ_0032463, miRNA 489 (miR-498), and *LEF1* using RNA pulldown, RNA immunoprecipitation (RIP), and luciferase assays. The effect of the circ_0032463/miR-498/LEF1 axis on the migration, proliferation, and apoptosis levels of OS cells was explored using CCK-8, bromodeoxyuridine (BrdU), wound healing, and fluorescein isothiocyanate (FITC) assays. Our findings revealed that circ_0032463 expression was upregulated in OS tissues and cell lines. We also found that circ_0032463 interacted with miR-498, thereby reducing the expression of miR-498 in OS cells. Experimental results indicated that miR-498 could directly target *LEF1* in OS cells and that circ_0032463 could abrogate the tumor-inhibitory effect of miR-498 by upregulating *LEF1* in OS. More specifically, by binding to miR-498 and inhibiting *LEF1* expression, circ_0032463 promoted the migration and proliferation abilities of OS cells and suppressed the apoptosis ability of OS cells. Overall, this research suggested that circ_0032463 could promote OS development by regulating the miR-498/LEF1 axis.

## INTRODUCTION

Osteosarcoma (OS) is a form of malignancy that originates from the cells found in the knees or at the end of other long bones, and it is typically common among young adults and adolescents (1). This cancer has a low 5-year survival rate, and it is treated using a combination of surgery and chemotherapy (2). Despite the use of these methods in treating OS patients, the recurrence and metastasis rates of OS are about 30 to 40% (2). By understanding the underlying mechanisms of OS growth and exploring novel biomarkers for OS diagnosis and treatment, the chances of survival of OS patients can be improved considerably.

Circular RNAs (circRNAs) are RNAs that form a closed-loop structure made of covalent bonds, and they are produced by the omission and addition of introns and exons (3). These genetic materials play pivotal roles in controlling and manipulating the translation and transcription of genes that affect cellular physiology and pathology (4). Evidence in several research articles revealed that by sponging specific microRNAs (miRNAs) and regulating the miRNAs’ targeted genes, circRNAs could regulate cell migration, invasion, proliferation, and regeneration in various cancers, such as gastric cancer (5), bladder cancer (6), and lung cancer (7). Besides, numerous studies have reported the impacts of circRNAs on regulating OS progression (8–10). For instance, Wu et al. found that circTADA2A promoted the growth and metastasis of OS (8). Zhang et al. also demonstrated that circ_0136666 predicted poor prognosis and initiated OS tumorigenesis (9). Even with these findings though, novel circRNAs involving OS development remain to be further explored to elaborate the pathogenesis of OS comprehensively and discover new biomarkers for OS diagnosis.

In contrast to circRNAs, microRNAs (miRNAs) are RNAs with noncoding abilities, and they can facilitate or decelerate the transcription of mRNAs by binding to specific target genes (11). Increasing evidence suggests that miRNAs play critical roles in regulating the growth of many cancer types, such as pancreatic cancer, cervical cancer, and lung cancer (12, 13). For instance, miRNA 489 (miR-498) suppressed lung cancer cells by targeting *HMGA2*, an oncogene (14). Another study reported that miR-498 inhibited the growth and metastasis of liver cancer cells by targeting ZEB2 (15). Yet, very little is known about the relationship between OS and the miR-498. Therefore, research efforts are needed to examine whether miR-498 can target circRNAs in OS.

The lymphoid enhancer-binding factor 1 gene (*LEF1*) is a protein-coding gene located on chromosome 4q25. Consisting of 13 exons, this gene belongs to a homologous family with high-mobility group protein 1 (16, 17). *LEF1* was demonstrated to participate in hair cell differentiation and follicle morphogenesis (18, 19). Recent research suggests that *LEF1* can promote cell growth and inhibit cell apoptosis in such cancers as colorectal cancer, lung cancer, and hepatocellular carcinoma (20–22). Research also revealed that *LEF1* acts as an oncogene in OS progression, thereby enhancing the migration and invasion proliferation abilities of OS cells (23–25). Nonetheless, the upstream molecules mediating LEF1 in the pathogenesis of OS have not been fully explored.

The aim of this paper was to examine how hsa_circ_0032463 (abbreviated “circ_0032463” here) initiates and regulates OS progression. We hypothesized that circ_0032463 could regulate the expression of *LEF1* by interacting with miR-498 to influence OS progression. Using several cellular experiments, we proved this hypothesis and demonstrated the potential value of circ_0032463/miR-498/LEF1 axis in the diagnosis and treatment of OS.

## RESULTS

### Identification of key regulators in OS.

**FIG 1:**
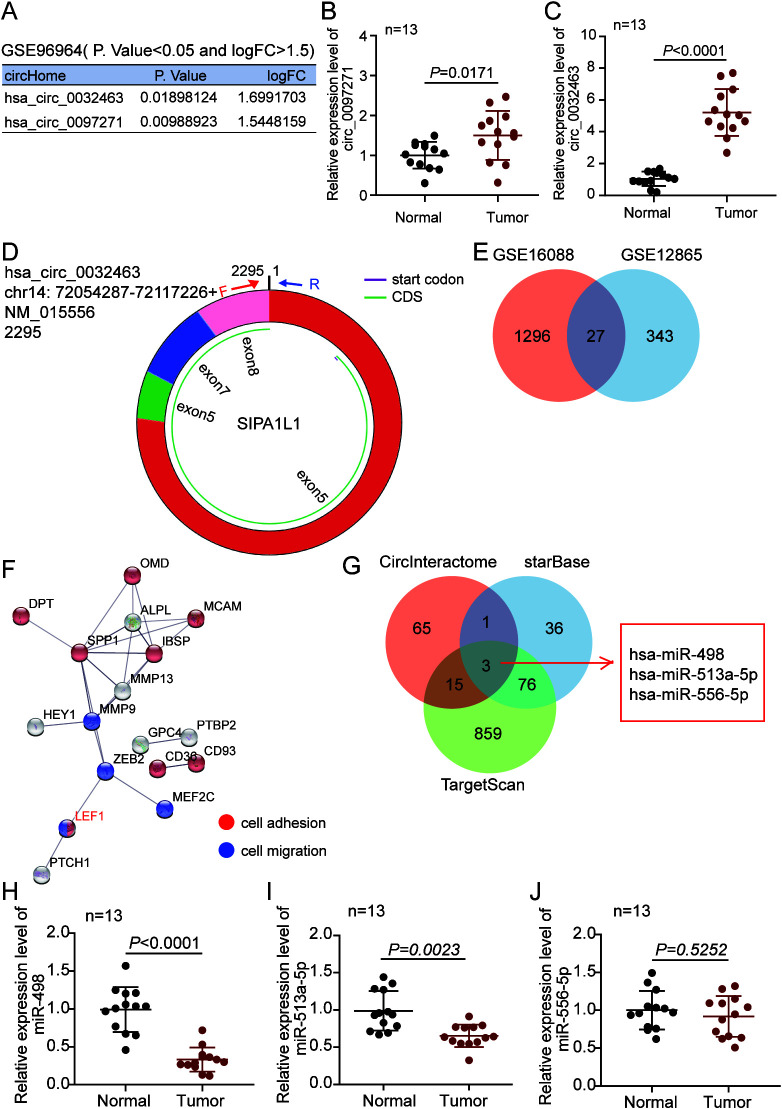
The circ_0032463/miR-498/LEF1 axis might act as a key regulatory pathway in osteosarcoma progression. (A) Two upregulated circRNAs were screened out by a *P* value of <0.05 and a logFC of >1.5 in a GEO data set, GSE96964. (B and C) RT-qPCR analysis revealed circ_0032463 expression in osteosarcoma tissues was higher than that in normal tissues. (D) The molecular structure of circ_0032463. (E) Venn diagram showing 27 upregulated genes overlapped from GSE16088 and GSE12865 based on an adj.*P* value of <0.05 and logFC of >2. (F) STRING analysis demonstrates *LEF1* as a key gene involving cell adhesion and cell migration among the 27 upregulated genes. (G) Venn diagram showing three miRNAs potentially interact with both circ_0032463 and LEF1. CircInteractome predicted the miRNAs binding to circ_0032463. TargetScan and starBase predicted the miRNAs binding to LEF1. (H to J) RT-qPCR analysis determined miR-498 was expressed lowest in osteosarcoma tissues among the three miRNAs.

To investigate the circRNAs involved in the pathogenesis of OS, we analyzed GSE96964 that was obtained from GEO DataSets. With an adjusted *P* value (adj.*P*) of <0.05 and log_2_ fold change (logFC) of >1.5, two upregulated circRNAs (circ_0032463 and circ_0097271) were identified (Fig. 1A). After performing reverse transcription-quantitative PCR (RT-qPCR) analysis, we noticed that the expression of circ_0032463, with a length of 2,295 bp, was higher than that of circ_0097271 in OS tissues (Fig. 1B and C). The copy number of circ_0032463 was higher than that of circ_0097271 in OS tissues (data not shown). Hence, circ_0032463 was confirmed as our circRNA of interest, and its structure is shown in Fig. 1D. To isolate the key genes participating in OS progression, two mRNA expression profiles (GSE16088 and GSE12865) were downloaded from the GEO DataSets. With an adj.*P* of <0.05 and logFC of >2, a total of 27 upregulated genes overlapped from GSE16088 and GSE12865 (Fig. 1E). After STRING analysis was performed, LEF1 was identified as a key molecule involved in cell adhesion and cell migration among the 27 upregulated genes (Fig. 1F). Next, three miRNAs (miR-498, miR-513a-5p, and miR-556-5p) were predicted to bind to both circ_0032463 and LEF1 by CircInteractome for the prediction of miRNAs binding to circ_0032463 and by starBase and TargetScan for the prediction of miRNAs binding to LEF1 (Fig. 1G). Finally, miR-498 was found with the lowest expression in OS tissues (Fig. 1H to J), and it was also found with the lowest copy number in OS tissues (data not shown). Therefore, miR-498 was identified as our miRNA of interest.

### Upregulation of circ_0032463 in OS cells.

**FIG 2:**
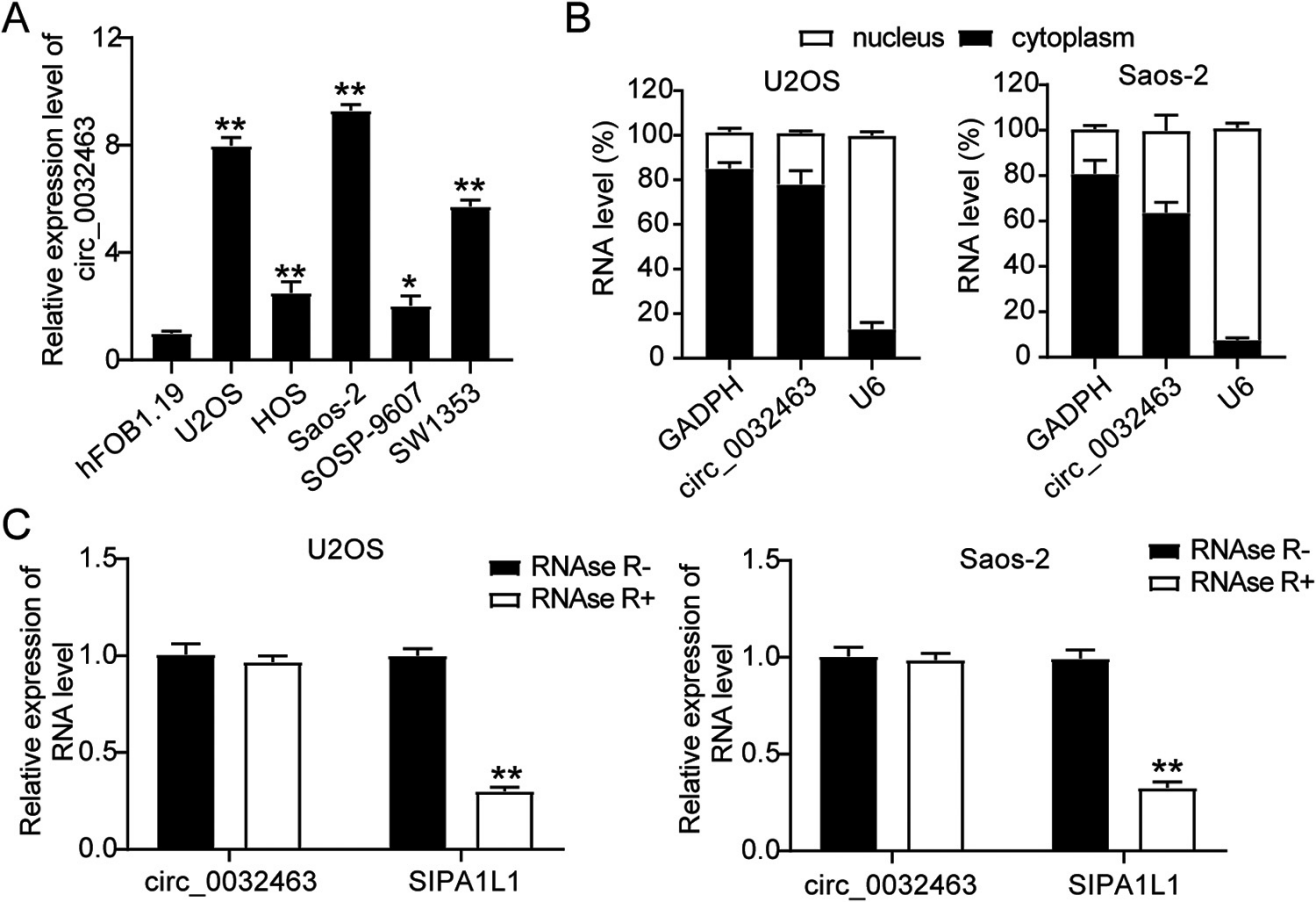
circ_0032463 is upregulated in osteosarcoma cells. (A) Measurement of circ_0032463 expression in osteosarcoma cell lines (U2OS, HOS, Saos-2, SOSP-9607, and SW1353) and the normal osteoblast cell line (hFOB1.19) by RT-qPCR. (B) Measurement of circ_0032463 expression in the nucleus and cytoplasm of U2OS and Saos-2 cells by RT-qPCR. (C) RT-qPCR analysis of circ_0032463 and SIPA1L1 mRNA after treatment with or without RNase R in U2OS and Saos-2 cells. *, *P* < 0.05; **, *P* < 0.001.

To investigate the potential function of circ_0032463 in OS progression, we examined the expression of circ_0032463 in five OS cell lines (SW1353, SOSP-9607, Saos-2, HOS, and U2OS) and a normal osteoblast cell line (hFOB1.19). The results revealed that the expression of circ_0032463 was significantly higher in the five OS cell lines than in hFOB1.19 cell lines. Moreover, U2OS and Saos-2 cells showed the highest circ_0032463 expression (Fig. 2A). Hence, we selected these two cell lines for further studies. Next, we isolated the nucleus and cytoplasm fraction of U2OS and Saos-2 cells to identify the circ_0032463 sublocalization in OS cells. The RT-qPCR results showed that circ_0032463 was mostly localized in the cytoplasm of U2OS and Saos-2 cells (Fig. 2B). Furthermore, after performing RNase R treatment assay, we discovered that RNase R treatment had no effect on the level of circ_0032463; however, it significantly decreased the level of linear RNA SIPA1L1 in U2OS and Saos-2 cells, meaning circ_0032463 existed in OS cells with a circular isoform (Fig. 2C). Collectively, these data suggested that circ_0032463 was significantly upregulated in OS cells and mainly located in the cytoplasm in a circular isoform.

### circ_0032463 promotes the malignancy of OS cells.

**FIG 3:**
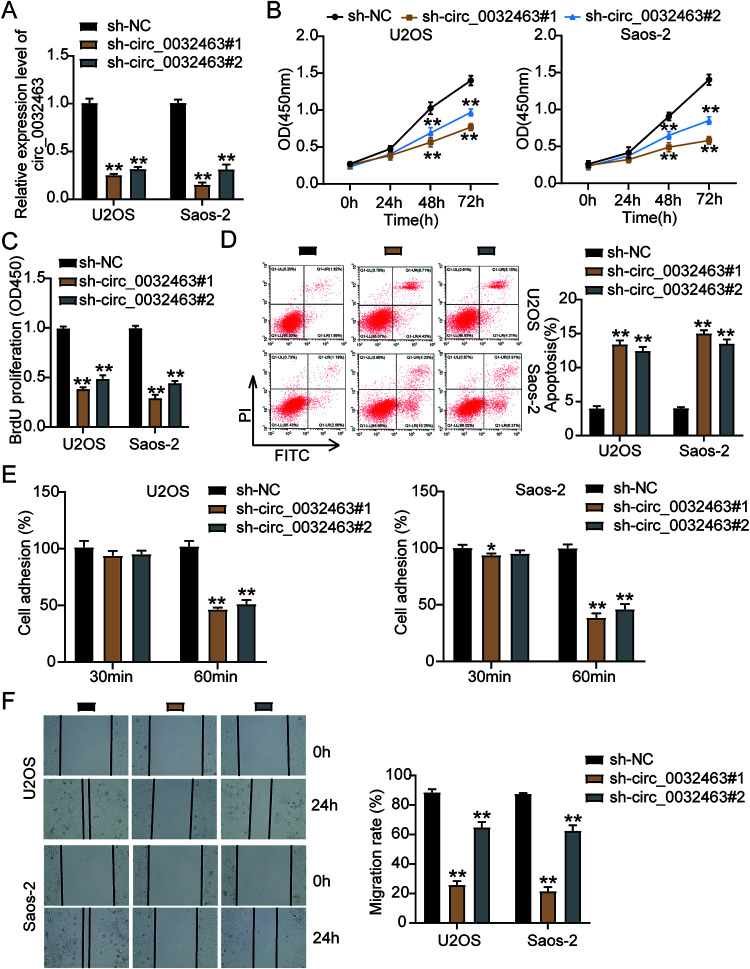
circ_0032463 promoted cell proliferation, adhesion, and migration but suppressed apoptosis of osteosarcoma cells. (A) RT-qPCR analysis of circ_0032463 level in U2OS and Saos-2 cells transfected with sh-NC, sh-circ_0032463#1, and sh-circ_0032463#2. (B) Cell viability was detected in U2OS and Saos-2 cells transfected with sh-NC, sh-circ_0032463#1, and sh-circ_0032463#2 at 0, 24, 48, and 72 h by CCK-8 assay. (C) Cell proliferation was detected in U2OS and Saos-2 cells transfected with sh-NC, sh-circ_0032463#1, and sh-circ_0032463#2 by BrdU assay. (D) Cell apoptosis was determined in U2OS and Saos-2 cells transfected with sh-NC, sh-circ_0032463#1, and sh-circ_0032463#2 by FITC apoptosis detection kit. (E) Cell adhesion was determined in U2OS and Saos-2 cells transfected with sh-NC, sh-circ_0032463#1, and sh-circ_0032463#2 at 30 and 60 min, respectively. (F) Cell migration ability was determined in U2OS and Saos-2 cells transfected with sh-NC, sh-circ_0032463#1, and sh-circ_0032463#2 by wound healing assay. sh-NC, shRNA-negative control; sh-circ_0032463#1 and sh-circ_0032463#2 are shRNAs of circ_0032463#1 and circ_0032463#2, respectively. *, *P* < 0.05; **, *P* < 0.001.

To further explore the potential biological impact of circ_0032463 on OS development and progression, we constructed two short hairpin RNAs (shRNAs) targeting circ_0032463 (sh-circ_0032463#1 and sh-circ_0032463#2) to genetically silence circ_0032463 in OS cell lines. After transfecting them into U2OS and Saos-2 cells, we evaluated the silencing efficiency using RT-qPCR. The results showed that the expression of circ_0032463 declined by 80% in sh-circ_0032463#1-transfected cells and by 70% in sh-circ_0032463#2-transfected cells (Fig. 3A). Based on the high silencing efficiency, we then performed a CCK-8 assay to measure the effect of circ_0032463 on OS cell viability. The results showed that the cell viability level of U2OS and Saos-2 cells transfected with sh-circ_0032463#1 or sh-circ_0032463#2 was lower than that of the cells transfected with the short hairpin RNA-negative control (sh-NC) (Fig. 3B). Consistently, the bromodeoxyuridine (BrdU) assay results showed a 50% reduction in the proliferation level of the U2OS and Saos-2 cells transfected with sh-circ_0032463#1 or sh-circ_0032463#2 compared with that in the cells transfected with sh-NC (Fig. 3C). At the same time, the flow cytometry findings revealed that compared with the sh-NC group cells, Saos-2 and U2OS cells transfected with sh-circ_0032463#1 or sh-circ_0032463#2 showed a 3-fold increase in cell apoptosis levels (Fig. 3D). Furthermore, after performing cell adhesion assay, we discovered a 50% decrease in cell adhesion level of Saos-2 and U2OS cells transfected with sh-circ_0032463#1 or sh-circ_0032463#2 compared to sh-NC group cells (Fig. 3E). Moreover, in the wound healing assay, U2OS and Saos-2 cells transfected with sh-circ_0032463#1 displayed a 65% decrease in the cell migration rate, while cells transfected with sh-circ_0032463#2 presented a 30% reduction in the cell migration rate compared to sh-NC group cells (Fig. 3F). Taken together, these results suggested that circ_0032463 could regulate OS progression. Meanwhile, sh-circ_0032463#1 was chosen for the next experiments as it had a stronger inhibitory effect on the malignancy of OS cells than sh-circ_0032463#2.

### circ_0032463 directly interacts with miR-498 in OS cells.

**FIG 4:**
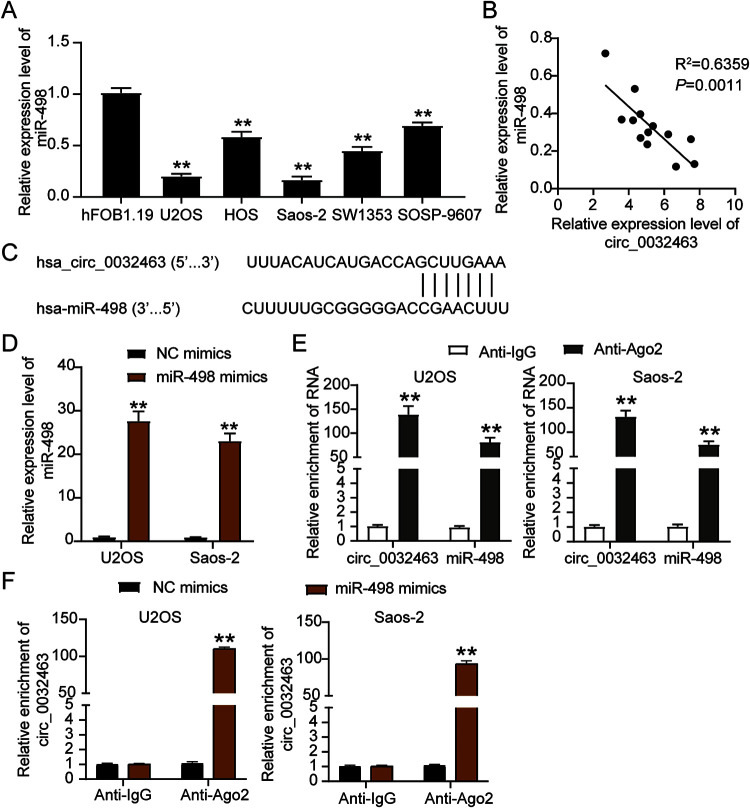
circ_0032463 directly interacted with miR-498 in osteosarcoma cells. (A) Measurement of miR-498 expression in osteosarcoma cell lines (U2OS, HOS, Saos-2, SOSP-9607, and SW1353) and the normal osteoblast cell line (hFOB1.19) by RT-qPCR. (B) The correlation between the relative expression level of miR-498 and circ_0032463 was evaluated by Pearson’s correlation test. (C) The potential binding sequences of miR-498 in circ_0032463 was predicted by CircInteractome. (D) RT-qPCR analysis of miR-498 expression in U2OS and Saos-2 cells transfected with NC mimics and miR-498 mimics. (E) The interaction of circ_0032463 and miR-498 was determined by RIP assay. (F) The enrichment of circ_0032463 in U2OS and Saos-2 cells transfected with NC mimics and miR-498 mimics was assessed by RIP assay. *, *P* < 0.05; **, *P* < 0.001. NC, negative control; Wt, wild type; Mut, mutant.

Bioinformatic analysis revealed that miR-498 was one of the key miRNAs that could interact with circ_0032463. Hence, it might act as a mediating factor in OS progression. We carried out a series of experiments to verify the relationship between circ_0032463 and miR-498. First, we performed RT-qPCR to observe the expression level of miR-498 in five OS cell lines (SW1353, SOSP-9607, Saos-2, HOS, and U2OS) and found that miR-498 expression in OS cell lines was lower than that in the hFOB1.19 cell line. Moreover, in the U2OS and Saos-2 cells, miR-498 expression was downregulated by 80% compared to expression in the hFOB1.19 cells (Fig. 4A). Moreover, the results of Pearson’s correlation analysis showed a negative correlation between the circ_0032463 level and miR-498 level in OS tissues (Fig. 4B), thus indicating the potential interaction between circ_0032463 and miR-498. Meanwhile, the prediction results of CircInteractome showed a potential binding site of miR-498in the sequence of circ_0032463 (Fig. 4C). We employed the luciferase reporter assay to further confirm the interaction between circ_0032463 and miR-498 and then transfected miR-498 mimics and negative-control (NC) mimics into U2OS and Saos-2 cells to assess the transfection efficiency. The RT-qPCR results revealed that compared to the expression of the NC mimic group, that of miR-498 in the cells transfected with miR-498 mimics was upregulated more than 20-fold (Fig. 4D). As for the results of the RNA immunoprecipitation (RIP) assay, circ_0032463 and miR-498 were abundantly enriched in Ago2 protein, meaning circ_0032463 interacted with miR-498 in both Saos-2 and U2OS cells (Fig. 4E and F). Overall, the above results revealed that circ_0032463 could directly interact with miR-498 in OS cells.

### Overexpression of circ_0032463 promotes the malignancy of osteosarcoma cells.

**FIG 5:**
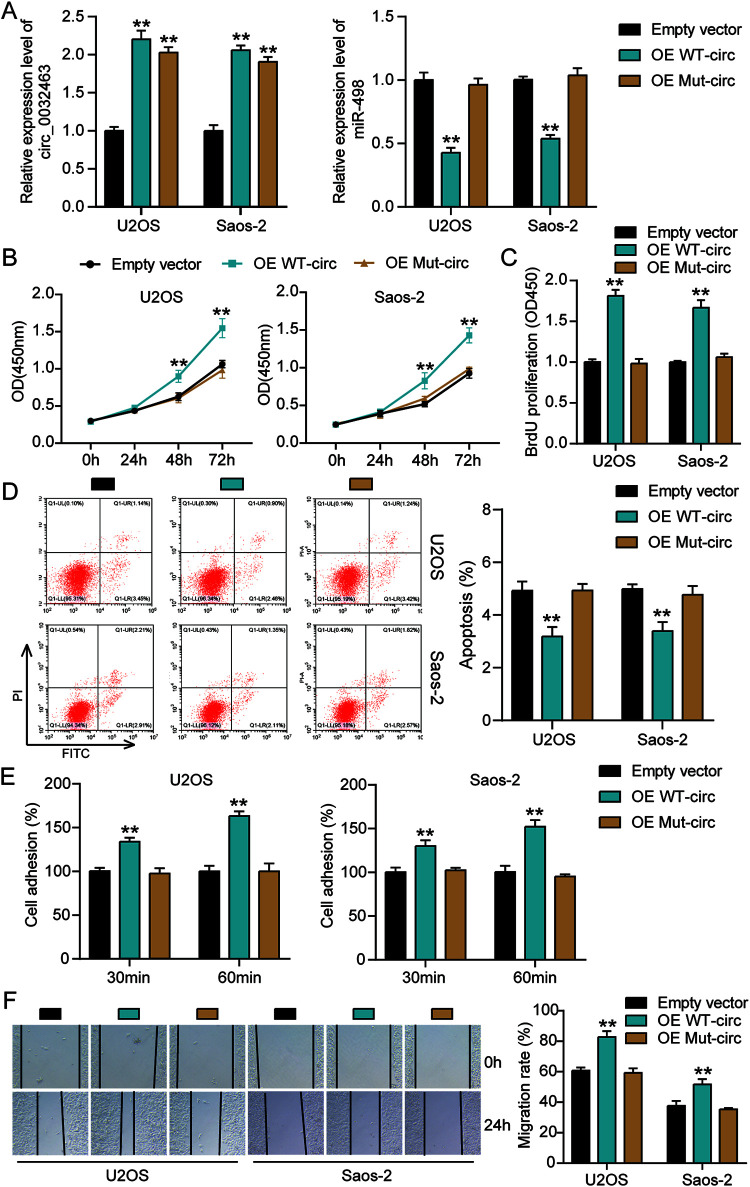
Overexpression of wild-type circ_0032463 but not circ_0032463 with mutant miR-498 binding sites promoted tumorigenesis in osteosarcoma cells. (A) Measurement of miR-498 and circ_0032463 expression in U2OS and Saos-2 cells transfected with empty vector, OE WT-circ, and OE Mut-circ by RT-qPCR. (B) Cell viability was detected in U2OS and Saos-2 cells transfected with empty vector, OE WT-circ, and OE Mut-circ. (C) Cell proliferation was detected in U2OS and Saos-2 cells transfected with empty vector, OE WT-circ, and OE Mut-circ. (D) Cell apoptosis was detected in U2OS and Saos-2 cells transfected with empty vector, OE WT-circ, and OE Mut-circ. (E) Cell adhesion was detected in U2OS and Saos-2 cells transfected with empty vector, OE WT-circ, and OE Mut-circ at 30 and 60 min. (F) Cell migration was detected in U2OS and Saos-2 cells transfected with empty vector, OE WT-circ, and OE Mut-circ. OE WT-circ, wild-type circ_0032463 overexpression vector; OE Mut-circ, circ_0032463 overexpression vector with mutant miR-498 binding sites. *, *P* < 0.05; **, *P* < 0.001.

To further investigate whether circ_0032463 regulates OS progression by interacting with miR-498, we constructed circ_0032463 overexpression vectors containing wild-type or mutant miR-498 binding sites and transfected them into U2OS and Saos-2 cells. The RT-qPCR was first carried out to examine the transfection efficiency, and the results indicated that the transfection of wild-type and mutant circ_0032463 upregulated the level of circ_0032463; however, only wild-type circ_0032463 decreased miR-498 expression in U2OS and Saos-2 cells (Fig. 5A). These results further confirmed that the predicted sequence was the binding sites of miR-498 in circ_0032463 and circ_0032463. The CCK-8 assay was performed, and the results showed a significant increase of cell viability in wild-type circ_0032463 overexpressed OS cells compared to that in the empty vector group of cells; nonetheless, the overexpression of mutant circ_0032463 had no influence on OS cells’ viability (Fig. 5B). Similarly, it was discovered that the proliferation of OS cells was enhanced by over 1.6-fold when wild-type but not mutant circ_0032463 was transfected (Fig. 5C). With regard to the flow cytometry results, the overexpression of wild-type circ_0032463 decreased the apoptosis rate of U2OS and Saos-2 cells by 20% compared with the empty vector group, while the overexpression of mutant circ_0032463 showed a comparable apoptosis rate to the empty vector group (Fig. 5D). The results of the cell adhesion assay and wound healing assay indicated that both the adhesion and migration abilities of OS cells were significantly enhanced by overexpression of wild-type circ_0032463; however, they were not affected by overexpression of mutant circ_0032463 (Fig. 5E and F). These data conclusively supported the view that circ_0032463 could regulate OS progression by interacting with miR-498.

### circ_0032463 accelerates OS tumorigenesis by inhibiting miR-498.

**FIG 6:**
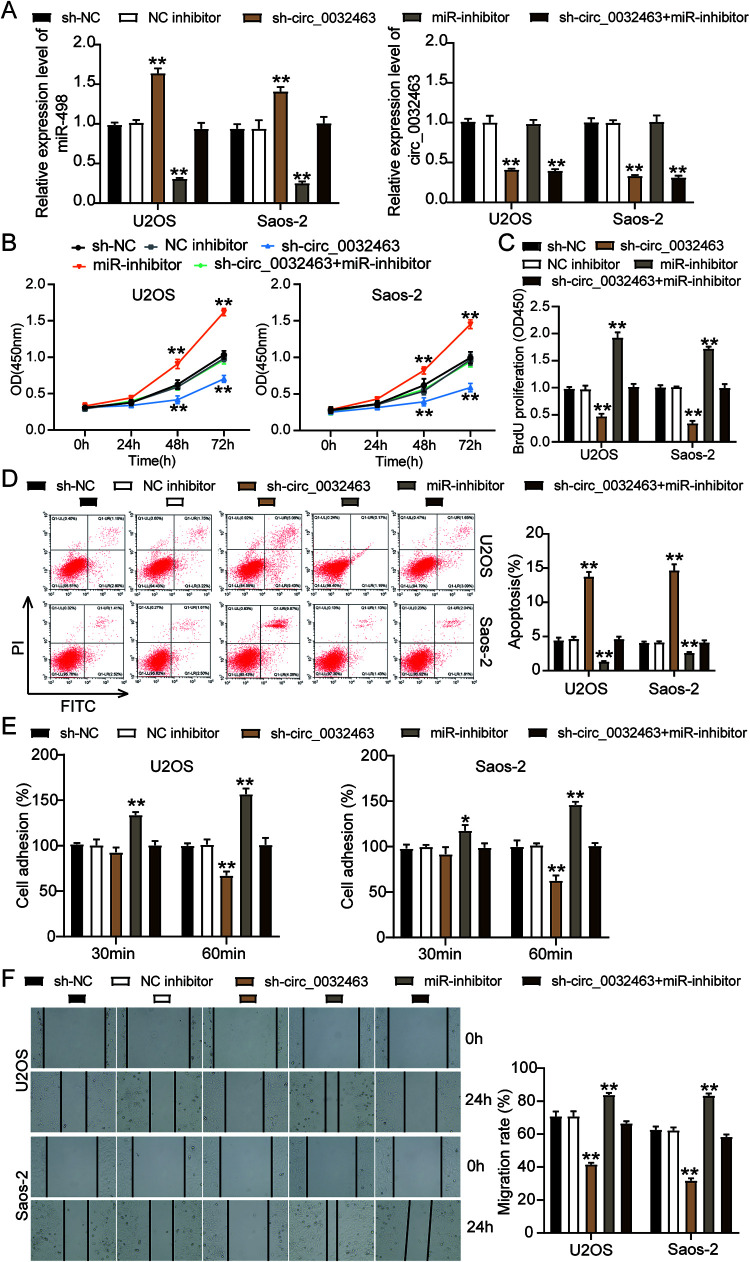
circ_0032463 accelerated the tumorigenesis in osteosarcoma cells by inhibiting miR-498. (A) Measurement of miR-498 and circ_0032463 expression in U2OS and Saos-2 cells transfected with sh-NC, NC inhibitor, sh-circ_0032463, miR-inhibitor, and sh-circ_0032463+miR-inhibitor by RT-qPCR. (B) Cell viability was detected in U2OS and Saos-2 cells transfected with sh-NC, NC inhibitor, sh-circ_0032463, miR-inhibitor, and sh-circ_0032463+miR-inhibitor by CCK-8 assay. (C) Cell proliferation was detected in U2OS and Saos-2 cells transfected with sh-NC, NC inhibitor, sh-circ_0032463, miR-inhibitor, and sh-circ_0032463+miR-inhibitor by BrdU assay. (D) Cell apoptosis was detected in U2OS and Saos-2 cells transfected with sh-NC, NC inhibitor, sh-circ_0032463, miR-inhibitor, and sh-circ_0032463+miR-inhibitor with the FITC apoptosis detection kit. (E) Cell adhesion was detected in U2OS and Saos-2 cells transfected with sh-NC, NC inhibitor, sh-circ_0032463, miR-inhibitor, and sh-circ_0032463+miR-inhibitor at 30 and 60 min. (F) Cell migration was detected in U2OS and Saos-2 cells transfected with sh-NC, NC inhibitor, sh-circ_0032463, miR-inhibitor, and sh-circ_0032463+miR-inhibitor by wound healing assay. sh-NC, shRNA-negative control; NC inhibitor, miR-498 inhibitor negative control; sh-circ_0032463, shRNA-circ_0032463; sh-circ_0032463+miR-inhibitor, shRNA-circ_0032463 plus miR-498 inhibitor. *, *P* < 0.05; **, *P* < 0.001.

To verify whether the inhibition of miR-498 could reverse the repressive impact of silencing circ_0032463 on OS cells, we constructed sh-circ_0032463, miR-498 inhibitor, and corresponding negative-control (NC) plasmids and then transfected them into Saos-2 and U2OS cells. The RT-qPCR results showed that the cells transfected with sh-circ_0032463 decreased the circ_0032463 level by 50% but elevated the miR-498 level by 1.5-fold compared to sh-NC cells. The OS cells transfected with the miR-498 inhibitor downregulated the miR-498 level by 60%, but no change in the circ_0032463 level was observed compared to sh-NC cells. At the same time, cells cotransfected with sh-circ_0032463 and the miR-498 inhibitor reduced the circ_0032463 level by 50%, while there was no change in the miR-498 level compared to sh-NC cells (Fig. 6A). The cell viability of OS cells transfected with the miR-498 inhibitor was higher than that of sh-NC cells; however, the cotransfection of sh-circ_0032463 and the miR-498 inhibitor counteracted the inhibitory effect of sh-circ_0032463 (Fig. 6B). Additionally, the BrdU assay results displayed that U2OS and Saos-2 cells transfected with the miR-498 inhibitor enhanced cell proliferation by 1.5-fold compared to sh-NC cells; however, this effect on cell proliferation could be reversed by cotransfecting sh-circ_0032463 and the miR-498 inhibitor (Fig. 6C). After carrying out flow cytometry, we found that OS cells transfected with the miR-498 inhibitor decreased cell apoptosis by 50% compared to sh-NC cells, and this effect could be counteracted by sh-circ_0032463 (Fig. 6D). Moreover, it was discovered that the proportion of adherent U2OS and Saos-2 cells was 1.5-fold enhanced when the miR-498 expression was inhibited. Moreover, the inhibitory effect of silencing circ_0032463 on OS cell adhesion was counteracted by inhibiting miR-498 (Fig. 6E). Likewise, the transfection of the miR-498 inhibitor increased the cell migration rate by 25%, in contrast to sh-NC cells, but it also could reverse the effect of transfecting sh-circ_0032463 on OS cell migration (Fig. 6F). In short, the above data indicated that circ_0032463 accelerated the tumorigenesis in OS cells by directly targeting and inhibiting miR-498.

### miR-498 directly targets LEF1 in OS cells.

**FIG 7:**
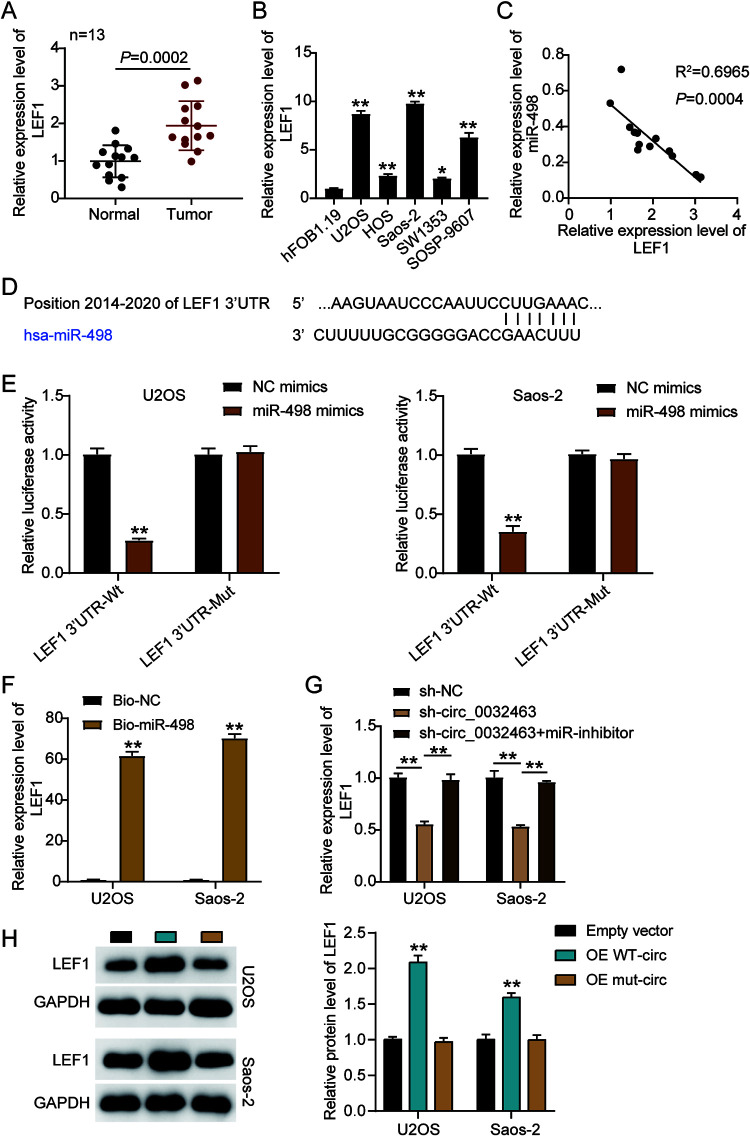
miR-498 was directly targeted with LEF1 in osteosarcoma cells. (A) RT-qPCR analysis of *LEF1* expression in osteosarcoma tissues (*n* = 13) and normal tissues (*n* = 13). (B) Measurement of *LEF1* expression in osteosarcoma cell lines (U2OS, HOS, Saos-2, SOSP-9607, and SW1353) and the normal osteoblast cell line (hFOB1.19) using RT-qPCR. (C) Pearson’s correlation analysis of the correlation between the relative expression level of miR-498 and LEF1. (D) The potential binding sequences of miR-498 on the LEF1 3′ UTR were predicted by TargetScan. (E) A dual-luciferase assay was performed to reveal the luciferase activity in cells cotransfected with plasmid LEF1 3′UTR-Wt or LEF1 3′UTR-Mut and NC mimics or miR-498 mimics. (F) The RNA pulldown assay determined the enrichment of LEF1 in U2OS and Saos-2 cells transfected with Bio-NC and Bio-miR-498. (G) RT-qPCR analysis of *LEF1* expression in U2OS and Saos-2 cells transfected with sh-NC, sh-circ_0032463, and sh-circ_0032463+miR-inhibitor. (H) Measurement of LEF1 protein level in U2OS and Saos-2 cells transfected with empty vector, OE WT-circ, and OE Mut-circ by Western blot assay. NC mimics, negative-control mimics; Wt, wild type; Mut, mutant; Bio-NC, biotin-negative control; Bio-miR-498, biotin–miR-498; sh-NC, shRNA-negative control; sh-circ_0032463, shRNA-circ_0032463; miR-inhibitor, miR-498 inhibitor; OE WT-circ, wild-type circ_0032463 overexpression vector; OE Mut-circ, circ_0032463 overexpression vector with mutant miR-498 binding sites. *, *P* < 0.05; **, *P* < 0.001.

We discovered that LEF1 could be upregulated in OS tissues and that it could interact with miR-498. Thus, we hypothesized miR-498 could directly target LEF1 to influence OS progression. To verify the relationship between miR-498 and LEF1, we first carried out RT-qPCR analysis and discovered that LEF1 expression was 2-fold higher in OS tissues than in corresponding normal tissues (Fig. 7A). We also observed that *LEF1* mRNA level was significantly upregulated in the five OS cell lines. In particular, Saos-2 and U2OS cells increased miR-498 expression by 10-fold compared to the level in hFOB1.19 cells (Fig. 7B). Moreover, as shown in Fig. 7C, a negative correlation between the expression of miR-498 and *LEF1* was found in OS tissues, suggesting that miR-498 might negatively regulate *LEF1* expression. Based on the predicted binding sequences of miR-498 on the 3′ untranslated region (UTR) of LEF1 (Fig. 7D), we constructed luciferase reporter plasmids (psiCHECK-2) containing the wild-type or mutant LEF1 3′ UTR and transfected them into U2OS and Saos-2 cells together with NC mimics or miR-498 mimics. The results showed that miR-498 overexpression could decrease the luciferase activity of U2OS and Saos-2 cells transfected with the psiCHECK-2 LEF1 3′ UTR wild-type vector (3′UTR-Wt) by about 60%, while it has no effect on the luciferase activity of the cells transfected with psiCHECK-2 LEF1 3′ UTR mutant vector (3′UTR-Mut), meaning that miR-498 could target and regulate LEF1 (Fig. 7E). To further confirm the interplay between LEF1 and miR-498, we performed an RNA pulldown assay in both the Saos-2 and U2OS cell lines and found that the enrichment of *LEF1* was increased in the biotin-labeled miR-498 mimics group compared to the NC mimics group, suggesting that miR-498 could directly interact with *LEF1* in OS cells (Fig. 7F). To further investigate whether circ_0032463 could mediate *LEF1* expression by regulating miR-498, we transfected sh-circ_0032463 and the miR-498 inhibitor into U2OS and Saos-2 cells. The RT-qPCR results revealed that cells transfected with sh-circ_0032463 presented a 50% decrease in *LEF1* expression, while there were no considerable changes in the cells cotransfected with sh-circ_0032463 and the miR-498 inhibitor compared with NC cells (Fig. 7G). Collectively, the data revealed that *LEF1* was a direct target gene of miR-498 in OS cells and that circ_0032463 could control *LEF1* expression by interacting with and restraining miR-498.

### miR-498 attenuates tumor development by repressing LEF1 in OS cells.

**FIG 8:**
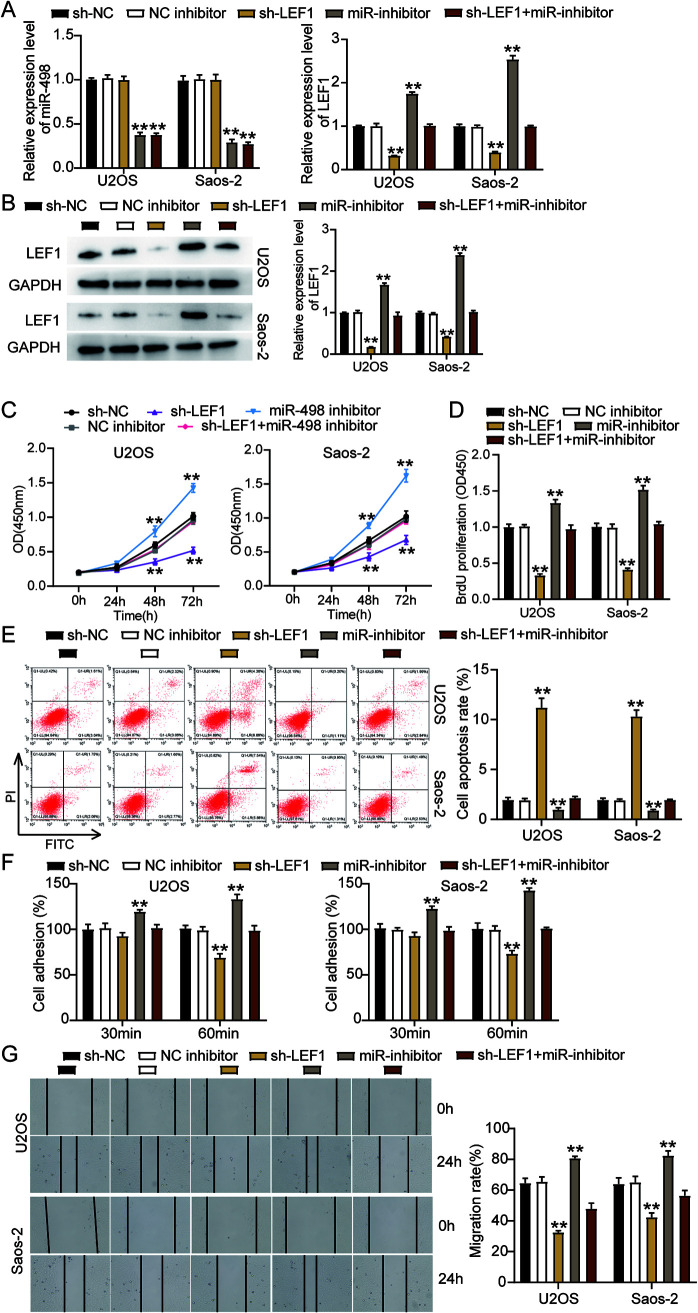
miR-498 attenuated tumor development by repressing LEF1 in osteosarcoma cells. (A) Measurement of miR-498 and *LEF1* expression in U2OS and Saos-2 cells transfected with sh-NC, NC inhibitor, sh-LEF1, miR-inhibitor, and sh-LEF1+miR-inhibitor by RT-qPCR. (B) Measurement of LEF1 protein expression in U2OS and Saos-2 cells transfected with sh-NC, NC inhibitor, sh-LEF1, miR-inhibitor, and sh-LEF1+miR-inhibitor by Western blot assay. (C) Cell viability was detected in U2OS and Saos-2 cells transfected with sh-NC, NC inhibitor, sh-LEF1, miR-inhibitor, and sh-LEF1+miR-inhibitor by CCK-8 assay. (D) Cell proliferation was detected in U2OS and Saos-2 cells transfected with sh-NC, NC inhibitor, sh-LEF1, miR-inhibitor, and sh-LEF1+miR-inhibitor by BrdU assay. (E) Cell apoptosis was detected in U2OS and Saos-2 cells transfected with sh-NC, NC inhibitor, sh-LEF1, miR-inhibitor, and sh-LEF1+miR-inhibitor with the FITC apoptosis detection kit. (F) Cell adhesion was detected in U2OS and Saos-2 cells transfected with sh-NC, NC inhibitor, sh-LEF1, miR-inhibitor, and sh-LEF1+miR-inhibitor at 30 and 60 min. (G) Cell migration was detected in U2OS and Saos-2 cells transfected with sh-NC, NC inhibitor, sh-LEF1, miR-inhibitor, and sh-LEF1+miR-inhibitor by wound healing assay. sh-NC, shRNA-negative control; NC inhibitor, negative control of miR-498 inhibitor; sh-LEF1, shRNA-LEF1; miR-inhibitor, miR-498 inhibitor; sh-LEF1+miR-inhibitor, shRNA-LEF1 plus miR-498 inhibitor. *, *P* < 0.05; **, *P* < 0.001.

We explored the impact of the interaction between miR-498 and LEF1 on OS progression by transfecting shRNA-LEF1, the miR-498 inhibitor, and the negative control (NC) into Saos-2 and U2OS cells. The results of RT-qPCR showed that the OS cells transfected with sh-LEF1 presented a reduction in *LEF1* expression by 70%, while no change was found in the miR-498 level compared to sh-NC cells. Meanwhile, cells transfected with the miR-498 inhibitor increased *LEF1* expression by 2-fold compared to sh-NC cells, while the effect was reversed following the transfection of the miR-498 inhibitor and sh-LEF1 (Fig. 8A). Besides, the cells transfected with sh-LEF1 reduced LEF1 protein expression by 50%, and the LEF1 protein level was upregulated by 2-fold, as observed in the cells transfected with the miR-498 inhibitor compared to sh-NC cells; however, the above effect on LEF1 expression was counteracted when the miR-498 inhibitor and sh-LEF1 were cotransfected (Fig. 8B). Subsequently, we observed that the cells transfected with sh-LEF1 inhibited U2OS and Saos-2 cell viability compared to the sh-NC cells, while the effect was abrogated when sh-LEF1 and the miR-498 inhibitor were cotransfected (Fig. 8C). Likewise, the BrdU assay results indicated that the proliferation ability of the cells transfected with sh-LEF1 was inhibited by 50%, in contrast to that in the sh-NC cells; however, this effect was abrogated by the cotransfection of the miR-498 inhibitor and sh-LEF1 (Fig. 8D). Furthermore, the flow cytometry results showed a 10-fold increase in the apoptosis rate of the cells transfected with sh-LEF1 compared to the sh-NC cells, while this promotive effect could be abrogated by the cotransfection of the miR-498 inhibitor and sh-LEF1 (Fig. 8E). At the same time, the cells transfected with sh-LEF1 suppressed the adhesion capacity of U2OS and Saos-2 cells by almost 30% compared with the sh-NC cells; however, this effect was neutralized by the miR-498 inhibitor (Fig. 8F). Similarly, transfection with sh-LEF1 suppressed the migration ability of U2OS and Saos-2 cells by 30% compared to that of the sh-NC cells, while this effect was reversed by cotransfecting the miR-498 inhibitor and sh-LEF1 (Fig. 8G). Collectively, these results clarified that miR-498 attenuated the development of OS cells by repressing LEF1.

### Overexpression of LEF1 reverses tumorigenesis suppression in OS cells.

**FIG 9:**
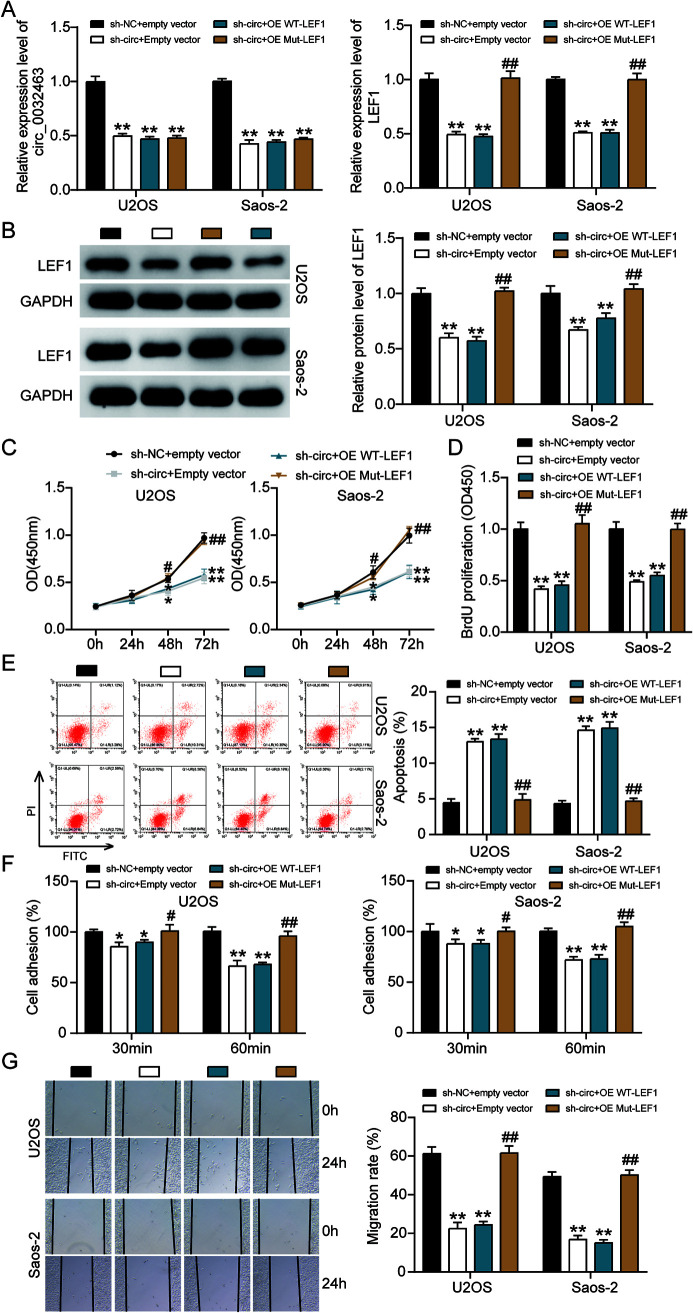
Overexpression of LEF1 with mutant but not wild-type miR-498 binding sites reversed the ameliorated tumorigenesis in circ_0032463-silenced osteosarcoma cells. (A) Measurement of circ_0032463 and LEF1 expression in U2OS and Saos-2 cells transfected with sh-NC+Empty vector, sh-circ+Empty vector, sh-circ+OE WT-LEF1, or sh-circ+OE Mut-LEF1 by RT-qPCR. (B) Measurement of LEF1 protein expression in U2OS and Saos-2 cells transfected with sh-NC+Empty vector, sh-circ+Empty vector, sh-circ+OE WT-LEF1, or sh-circ+OE Mut-LEF1 by Western blot assay. (C) Cell viability was detected in U2OS and Saos-2 cells transfected with sh-NC+Empty vector, sh-circ+Empty vector, sh-circ+OE WT-LEF1, and sh-circ+OE Mut-LEF1. (D) Cell proliferation was detected in U2OS and Saos-2 cells transfected with sh-NC+Empty vector, sh-circ+Empty vector, sh-circ+OE WT-LEF1, or sh-circ+OE Mut-LEF1 by BrdU assay. (E) Cell apoptosis was detected in U2OS and Saos-2 cells transfected with sh-NC+Empty vector, sh-circ+Empty vector, sh-circ+OE WT-LEF1, or sh-circ+OE Mut-LEF1 by FITC assay. (F) Cell adhesion was detected in U2OS and Saos-2 cells transfected with sh-NC+Empty vector, sh-circ+Empty vector, sh-circ+OE WT-LEF1, or sh-circ+OE Mut-LEF1 at 30 and 60 min. (G) Cell migration was detected in U2OS and Saos-2 cells transfected with sh-NC+Empty vector, sh-circ+Empty vector, sh-circ+OE WT-LEF1, sh-circ+OE Mut-LEF1 by wound healing assay. sh-NC+Empty vector, shRNA-negative control plus empty vector; sh-circ+Empty vector, shRNA-circ_0032463 plus empty vector; sh-circ+OE WT-LEF1, shRNA-circ_0032463 plus wild-type circ_0032463 overexpression vector; sh-circ+OE Mut-LEF1, shRNA-circ_0032463 plus circ_0032463 overexpression vector with mutant miR-498 binding sites. *, *P* < 0.05, and **, *P* < 0.001, versus the sh-NC+Empty vector group; ^#^, *P* < 0.05, and ^##^, *P* < 0.001, versus the sh-circ+Empty vector group.

To further investigate whether circ_0032463 could regulate OS progression via the miR-498/LEF1 axis, we constructed LEF1 overexpression vectors containing wild-type or mutant miR-498 binding sites and transfected them into U2OS and Saos-2 cells together with sh-circ_0032463 or sh-NC. RT-qPCR analysis was then performed to examine the levels of circ_0032463 and *LEF1*. The results showed that circ_0032463 expression was reduced by about 50% in the cells transfected with sh-circ_0032463 and wild-type LEF1, sh-circ_0032463 and mutant LEF1, and sh-circ_0032463 and empty vector. The results indicated that LEF1 overexpression had no regulatory effect on circ_0032463 expression. Regarding *LEF1* expression, the data showed that circ_0032463 silence repressed *LEF1* mRNA expression by ∼50%, while the cotransfection of mutant but not wild-type LEF1 vector reversed the downregulation of LEF1 in U2OS and Saos-2 cells with sh-circ_0032463 (Fig. 9A). The Western blot assay results revealed a consistent tendency of LEF1 protein expression to be associated with its mRNA expression in each cell group (Fig. 9B). These results indicated that circ_0032463 could regulate LEF1 expression by mediating the interaction between miR-498 and LEF1. After the CCK-8 assay was conducted, we discovered that overexpression of mutant LEF1 could significantly overcome the limitation of OS cell viability caused by the silence of circ_0032463. However, the overexpression of wild-type LEF1 did not have this impact (Fig. 9C). We also observed that the reduced proliferation level of OS cells, which was induced by silencing circ_0032463, was restored by overexpressing mutant but not wild-type LEF1 (Fig. 9B). In addition, the flow cytometry assay results revealed that overexpression of mutant LEF1 reversed the upregulated cell apoptosis elicited by silencing circ_0032463 both in U2OS and Saos-2 cells, while OS cells with the overexpression of mutant LEF1 showed a comparable apoptosis rate to the cells transfected with sh-circ_0032463 and the empty vector group (Fig. 9D). Furthermore, the results of the cell adhesion assay and wound healing assay showed that that the adhesion and migration abilities of OS cells were remarkably suppressed in the cells transfected with sh-circ_0032463; however, this suppressive effect could be totally reversed by overexpression of mutant LEF1, while not being affected by overexpression of wild-type LEF1 (Fig. 5E and F). These data collectively indicated that circ_0032463 could participate in the pathogenesis of OS by interacting with miR-498 to regulate target gene *LEF1*.

## DISCUSSION

In this study, we demonstrated that circ_0032463 was upregulated in OS tissues and cell lines. We also discovered that circ_0032463 promoted the migration and proliferation abilities of OS cells but suppressed the apoptosis ability of OS cells by directly targeting miR-498 and enhancing LEF1 expression. Based on this, we suggested that circ_0032463 should be considered a novel biomarker for treating and diagnosing OS.

In recent years, evidence in the literature has confirmed that circRNAs have the ability to fuel the growth of various forms of cancers. The interaction of circRNAs with miRNAs was indicated to participate in cell migration, proliferation, and apoptosis. For instance, Xiang et al. demonstrated that circular RNAs (circCCDC66) contributed to OS development by regulating miR-338-3p and its associated protein, PTP1B (26). Zhang et al. also indicated that circ_0095424 regulated cells’ ability to migrate, regenerate, and proliferate by controlling the phosphatidylinositol 3-kinase (PI3K)/AKT signaling pathway through the miR-1238/HMGB1 axis (27). Similar to the findings of Xiang et al., Gu et al. revealed that circFAT1(e2) promoted OS growth by regulating miR-181b and HK2 (28). In this study, we discovered that circ_0032463, a new circRNA originating from its host gene, *SIPA1L1*, was upregulated in OS tissues and cell lines. Furthermore, we found that circ_0032463 silence inhibited OS cells’ migration and proliferation abilities, while it promoted their apoptosis ability. This finding improved our knowledge of the role of circRNA in OS progression.

Previous pieces of research have confirmed that miRNAs could influence cellular growth and apoptosis in numerous types of cancers, such as colorectal cancer, prostate cancer, and esophageal squamous cell carcinoma (29–31). Evidence was also found that miR-106b could promote the invasion and spread of malignancy by enhancing EMT (epithelial-mesenchymal transition) and downregulating Smad 7 (29). In several studies, miR-498 has been linked to cancer progression. One study, for instance, revealed that miR-498 inhibited cancerous growth in the lungs by targeting *HMGA2*, an oncogene (14). Another study uncovered that miR-498 inhibited cell growth by regulating ZEB2 in patients with liver cancer (15). These studies suggested that miR-498 limited cancerous growth by regulating cell proliferation and apoptosis. Similarly, our experimental results indicated that the miR-498 expression level was downregulated in OS tissues and cell lines and that miR-498 had a suppressive impact on OS cells’ proliferation, migration, and adhesion abilities, while it showed a promotive effect on OS cells’ apoptosis rate. Furthermore, we demonstrated that the inhibition of miR-498 could reverse the aggressive influence of circ_0032463 on OS malignancy. These findings highlighted the importance of the interaction between circ_0032463 and miR-498 in OS progression.

Furthermore, a growing body of evidence suggested that *LEF1* acted as an oncogene in various forms of malignant tumors, including colorectal cancer, lung cancer, and hepatocellular carcinoma (20–22). For example, by directly activating the NOTCH pathway, LEF1 overexpression promoted poor differentiation and progression of hepatocellular carcinoma (20). Lu et al. demonstrated that LEF1 overexpression boosted the invasion, migration, and proliferation abilities of OS cells. They also found that long noncoding RNA (lncRNA) LEF1‐AS1 sponged HNRNPL to elevate the expression of LEF1 and facilitate OS development (23). Additionally, Chen et al. indicated that LEF1 demonstration was upregulated in OS cells. Besides, circMYO10 promoted OS progression by facilitating the transcriptional activity of the β-catenin/LEF1 complex (25). These results suggested *LEF1* functioned as an oncogene in OS cells. Similar to the findings of previous studies, our results revealed that LEF1 expression level was evidently upregulated in OS tissues and cell lines and that it boosted OS cells’ migration and proliferation but repressed OS cells’ apoptosis rate. However, in contrast to the results of previous studies, we proved that miR-498 was the upstream miRNA to target LEF1 and that LEF1 expression could be regulated by circ_0032463.

In previous studies, LEF1 dysregulation has been involved in various signaling pathways. For instance, LEF1 mediated osteoarthritis progression via the NF-κB signaling pathway (32). Also, miR-34a-5p restricted the invasion, migration, and epithelial-mesenchymal transition level of cells with esophageal cancer, not only by inactivating the Hippo-YAP1/TAZ signaling pathway but also by targeting LEF1 (33). These studies suggested that the NF-кB signaling pathway and Hippo-YAP1/TAZ signaling pathway might be associated with LEF1 in the development of cancers. The effects of these pathways on OS cells should be further explored in the future. Besides, more experiments should be performed in animal models to confirm the impacts of the circ_0032463/miR-498/LEF1 axis on cells with OS.

In sum, our study confirmed that circ_0032463 facilitated the migration and proliferation abilities of OS cells but that it restricted the apoptosis ability of OS cells via the miR-498/LEF1 axis. The knowledge of this research could provide potential biomarkers and crucial insights into OS treatments.

## MATERIALS AND METHODS

### Clinical samples and cell lines.

Tumor tissue specimens (*n* = 13) and corresponding adjacent normal tissues (*n* = 13) were obtained from OS patients at the First Affiliated Hospital of Zhengzhou University. Informed written consent was provided by all the participants, and all procedures used in this study were approved by the Ethics Committee of the First Affiliated Hospital of Zhengzhou University. The clinical information of the 13 OS patients is listed in Table 1. Five human OS cell lines (SOSP-9607, Saos-2, HOS, SW1353, and U2OS) and a normal osteoblast cell line (hFOB1.19) were purchased from ATCC (Manassas, VA, USA). The hFOB1.19 cell line was cultured in a Dulbecco's modified Eagle’s medium (DMEM), U2OS and Saos-2 cells in a McCoy’s 5A medium, SW1353 cells in an L-15 medium, HOS cells in MEM, and SOSP-9607 cells in an RPMI 1640 medium. All of the culture media were supplemented with 10% fetal bovine serum (FBS), and all the cell lines were maintained in a space containing 5% CO_2_ at 37°C. All reagents were purchased from Life Technologies (Gibco, Waltham, MA, USA).

**TABLE 1:** Clinical characteristics of 13 osteosarcoma patients

Characteristic	No. (%) of patients
Total	13
Gender	
Male	7 (53.85)
Female	6 (46.15)
Age (yr)	
<12	1 (7.69)
12–29	7 (53.85)
30–49	3 (23.08)
≥50	2 (15.38)
Tumor size (cm)	
≤12	8 (61.54)
>12	5 (38.46)
TNM stage	
I–II	10 (76.92)
III–IV	3 (23.08)
Site	
Extremities	9 (69.23)
Other	4 (30.77)
Distant metastasis	
Negative	9 (69.23)
Positive	4 (30.77)

### RT-qPCR and subcellular location of circRNA.

The total RNA from clinical tissues and cultured cells was extracted with TRIzol reagent (Sigma, St. Louis, MO, USA). This extraction was performed according to the manufacturer’s instructions. For mRNA detection, the extracted total RNA was transcribed into cDNA using the PrimeScript First Strand cDNA synthesis kit (RR037A; TaKaRa, Shiga, Japan), and the RT-qPCR was conducted with SYBR Premix *Ex Taq* (RR420A; TaKaRa, Japan). The quantification of circRNA was the same as that of mRNA; however, the RNase R treatment was employed to remove linear RNA before transcription. For miRNA, the total RNA from cultured cells and clinical tissues was extracted using the miRcute miRNA isolation kit (DP501; Tiangen Biotech, Beijing, China), and it was transcribed into cDNA with the miRcute miRNA First-strand cDNA synthesis kit (KR211; Tiangen Biotech, China). RT-qPCR was carried out with the miRcute Enhanced miRNA fluorescence quantitative detection kit (FP411; Tiangen, Beijing, China). Gene expression was computed using the threshold cycle (2^−ΔΔ^*^CT^*) method with GAPDH (glyceraldehyde-3-phosphate dehydrogenase) serving as the reference control of mRNA and circRNA, while U6 functioned as the reference control of miRNA. All of the primers utilized in this research are listed in Table 2. RT-qPCR analysis was performed to identify the subcellular localization of circRNA. The PARIS kit (Life Technologies, USA) was then used to isolate cytoplasmic and nuclear fractions according to the manufacturer’s guidelines. Finally, the circRNA expression in these fractions was identified using RT-qPCR.

**TABLE 2:** Primer sequences for qRT-PCR

Gene	Direction	Primer sequence
circ_0032463	Forward	5′-GCCCAGCCTTTGAAGAATTT-3′
	Reverse	5′-TTCTGTGGTTGCCATAATCC-3′
circ_0097271	Forward	5′-CTGTGGAAACCCTTGGTTGT-3′
	Reverse	5′-TCACCTGTGAGAATTGACTGG-3′
miR-498	Forward	5′-GGTTTGAAGCCAGGCGGTTTC-3′
	Reverse	5′-CAGTGCAGGGTCCGAGGTAT-3′
miR-513a-5p	Forward	5′-TGCGCTCAGCAAACATTTATTG-3′
	Reverse	5′-CCAGTGCAGGGTCCGAGGTATT-3′
miR-556-5p	Forward	5′-GCCGAGGATGAGCTCATTGT-3′
	Reverse	5′-CTCAACTGGTGTCGTGGA-3′
LEF1	Forward	5′-GTACAGCCTTCTCACGCAGT-3′
	Reverse	5′-GAAAACCAGCCAAGAGGTGG-3′
GAPDH	Forward	5′-TGCATCCTGCACCACCAACT-3′
	Reverse	5′-TGCCTGCTTCACCACCTTC-3′
U6	Forward	5′-CTCGCTTCGGCAGCACA-3′
	Reverse	5′-AACGCTTCACGAATTTGCGT-3′

### Evaluation of circRNA stability.

The RNase R treatment assay was performed to evaluate the stability of circRNA. Briefly, 1 μg of total RNA was treated with 3 U/μg RNase R (Epicentre Technologies, USA) for 30 min at 37°C. After that, it was purified with the Qiangen-manufactured RNeasy MinElute Cleanup kit (74204; Qiangen, Germany). The purification was done according to the manufacturer’s instructions. After transcription, the expression of circRNA and its linear gene, *SIPA1LA*, was detected by RT-qPCR.

### Cell transfection.

All biological materials required for cell transfection were purchased from RiboBio Co., Ltd. (China), such as sh-LEF1, negative-control (NC) inhibitors, miR-498 inhibitors, negative-control (NC) mimics, miR-498 mimics, sh-NC, sh-circ_0032463#2, sh-circ_0032463#1, pcDNA 3.1 empty vectors, pcDNA3.1 vectors containing wild-type or mutant circ_0032463, and pcDNA3.1 vectors containing wild-type or mutant LEF1. For cell transfection, Saos-2 and U2OS cells were harvested, and 24-well plates were seeded with them at a density of 2 × 10^5^ cells/well. After culture for 12 h, the vectors were transfected into the two cell lines for 48 h with Invitrogen-manufactured Lipofectamine 3000 (USA). Finally, the cells were harvested and used for subsequent experimental assays.

### Western blot assay.

The transfected U2OS and Saos-2 cells were treated with radioimmunoprecipitation assay (RIPA) buffer (Solarbio, China) containing a 1% proteinase inhibitor cocktail. After that, the proteins were extracted and quantified with the Pierce bicinchoninic acid (BCA) protein assay kit (Thermo Fisher Scientific, USA). Subsequently, 10% SDS-PAGE-based electrophoresis was carried out to separate the proteins. Next, they were electronically transferred to polyvinylidene difluoride (PVDF) membranes (Millipore, USA) at 72 V for 2 h. After that, 5% nonfat milk buffer was used to block the membranes at room temperature for 2 h. The anti-LEF1 (1:1,000, ab137872; Abcam, United Kingdom) and anti-GAPDH (1:1,000, ab9485; Abcam, United Kingdom) primary antibodies were then prepared and added to the membranes. The mixture was later incubated overnight at 4°C. Subsequently, the horseradish peroxidase (HRP)-conjugated anti-rabbit secondary antibody (1:1,000, ab6721; Abcam, United Kingdom) was prepared and added, and the mixture was incubated for 1 h at room temperature. The membrane was then washed with phosphate-buffered saline (PBS) three times, and the protein bands were detected using ECL enhanced chemiluminescence Western blotting detection reagents (Beyotime, China). ImageJ software was finally used to calculate the density of the blots.

### CCK-8 assay.

The transfected U2OS and Saos-2 cells were cultured in 96-well plates at a density of 5 × 10^3^ cells/well. After culture for 0, 24, 48, and 72 h, CCK-8 solution (catalog no. K1018; APExBIO, China) was added to each cell well, and the mixture was incubated for another 1.5 h. This procedure was done according to the manufacturer's protocol. Finally, the optical density at 450 nm in each cell well was detected with a multimode reader (Tecan, Männedorf, Switzerland).

### BrdU assay.

The BrdU assay was performed with the BrdU cell proliferation enzyme-linked immunosorbent assay (ELISA) kit (catalog no. ab126556; Abcam, USA). First, 96-well plates were seeded with the transfected U2OS and Saos-2 cells and cultured (density of 5 × 10^3^ cells/well). After cells reached a density of 70%, they were treated with 20 μl diluted 1× BrdU label solution at 37°C for 12 h. After the culture medium was aspirated from the cell wells, fixing solution (200 μl/well) was added, and the mixture was incubated at room temperature for 30 min to fix the cells and denature the DNA. Next, the plates were washed with 1× wash buffer three times. After that, 100 μl/well anti-BrdU monoclonal detector antibody was added to the cells, and the mixture was incubated for 1 h. After the unbound antibody was cleaned with wash buffer, 100 μl 1× peroxidase-conjugated goat anti-mouse IgG and 100 μl 3,3′,5,5′-tetramethylbenzidine (TMB) peroxidase substrate were consecutively pipetted into each cell well, and the mixture was incubated for 30 min at room temperature. Finally, the reaction was stopped with 100 μl stop solution, and the optical density at 450 nm was measured with a plate reader.

### Apoptosis assay.

The annexin V apoptosis detection kit (556547; BD, USA) was used in the apoptosis assay based on the manufacturer’s guidelines. First, transfected Saos-2 and U2OS cells (6 × 10^5^) were collected in a tube (1.5 ml), centrifuged, and then suspended with 100 μl binding buffer, which was comprised of 5 μl annexin V-fluorescein isothiocyanate (FITC) and 5 μl propidium iodide (PI). After that, the samples were incubated for 15 min at 4°C in the absence of light. Subsequently, the binding buffer (400 μl) was added to the cells to remove the redundant dye. After the cells were washed twice, the characteristics of the cells were identified with flow cytometry. The results of the cell apoptosis were analyzed using FlowJo (Tree Star, Ashland, OR, USA). The sum of the upper-right quadrant and lower-right quadrant represented the total cell apoptosis rate.

### Cell adhesion assay.

Twelve-well plates were seeded with the transfected U2OS and Saos-2 cells. After the cells adhered to dishes, the culture media were changed to serum-free media, and the cells were cultured for 8 h at 37°C. After that, they were digested with trypsin containing 10 mM EDTA. Next, 100 μl cell suspension containing 1 × 10^5^ cells was moved to the cell adhesion plate, which was precoated with collagen I solution (catalog no. C7661; Sigma, USA). The samples were subsequently incubated at 37°C for 30 or 60 min. After that, the cells that did not adhere to the plate were gently removed with PBS. The next step was the incubation of the cells for 2 h with 10 μl of MTT [3-(4,5-dimethyl-2-thiazolyl)-2,5-diphenyl-2H-tetrazolium bromide] substrate (catalog no. CT01; Sigma, USA). Eventually, 100 μl dimethyl sulfoxide (DMSO) was added to cells, and the optical density at 570 nm was determined using a multimode-plate-reader (Tecan, Männedorf, Switzerland).

### Cell wound healing assay.

The cell wound healing assay was performed to determine the cell migration ability of OS cells. First, 12-well plates were seeded with transfected U2OS and Saos-2 cells at 3 × 10^6^ cells/well, and the cells were incubated for 24 h. Next, wounds were created by scratching the center of the fused cell monolayer with a 200-μl micropipette tip. The exfoliated cells were gently removed by washing them with PBS. The cells were then cultured in a serum-free medium for 24 h. The images of the wound at 0 and 24 h were captured under a light microscope. Finally, the width of the wound at 0 and 24 h was measured. The migration rate was finally calculated using the following formula: [(width at 0 h − width at 24 h)/width at 0 h] × 100%.

### RNA immunoprecipitation assay.

The RIP assay was conducted to unravel the endogenous interaction of circ_0032463 and miR-498. Briefly, U2OS and Saos-2 cells transfected with miR-498 mimics or NC mimics were collected and lysed with the lysis buffer (catalog no. 20-188; Sigma, USA). Then the cell lysates were incubated at 4°C for 4 h with IgG (catalog no. ab172730; Abcam, United Kingdom) or Ago2 (catalog no. ab186733; Abcam, United Kingdom). Subsequently, the prepared protein A magnetic beads (catalog no. ab214286; Abcam, United Kingdom) were added to the treated cell lysates, and the lysates were incubated for 2 h to bind with the IgG. Finally, the enrichment of circ_0032463 and miR-498 on the beads was eluted and evaluated using an RT-qPCR system.

### Dual-luciferase reporter assay.

The materials for the dual-luciferase assay were purchased from Tuoran Bio (Shanghai, China), such as the psiCHECK2 LEF1 3′UTR-Mut vector and the psiCHECK2 LEF1 3′UTR-Wt vector. Lipofectamine 3000 transfection reagent was utilized to transfect Saos-2 and U2OS cells in the 24-well plate together with miR-498 mimics or NC mimics. After 48 h, the cells were harvested and subjected to detect the activities of firefly and *Renilla* luciferase using a dual-luciferase reporter assay system (E1910; Promega, USA). Relative luciferase activity was eventually calculated by normalizing the firefly luciferase activity to *Renilla* luciferase activity.

### RNA pulldown assay.

In this assay, U2OS and Saos-2 cells (3 × 10^5^) were first transfected with the biotin-labeled miR-490-3p (Bio-miR-490-3p) or miR-NC (Bio-NC), which was bought from RiboBio (China). After 48 h, the cells were collected, lysed with lysis buffer, and incubated with streptavidin beads (Thermo Scientific Fisher, USA) for 2 h at room temperature. The beads were then collected and washed with PBS twice. Finally, the RNAs attached to the beads were eluted and cleaned with the RNeasy minikit (74104; Qiagen, Germany). Finally, RT-qPCR analysis was performed to evaluate the abundance of LEF1.

### Ethics approval and consent to participate.

The present study was approved by the Ethics Committee of the First Affiliated Hospital of Zhengzhou University (Zhengzhou, China). The processing of clinical tissue samples was performed in strict compliance with the ethical standards of the Declaration of Helsinki. All patients signed written informed consent. Consent for publication was obtained from the participants.

### Statistical analysis.

Statistical analyses performed in this project included Wilcoxon two-tailed paired *t* test and one-way analysis of variance (ANOVA) with Dunnett’s *post hoc* test. The former was employed to test the statistical difference between two variables, while the latter was leveraged to evaluate the statistical difference among multiple groups. GraphPad Prism 8.0 was the software used to perform this statistical analysis. Three independent tests were performed, and all of the data were derived in the form of mean ± standard deviation (SD). The null hypothesis was rejected if *P* values were less than 0.05, meaning the values were assumed to be statistically significant.

### Availability of data and materials.

The data sets used and/or analyzed during the current study are available from the corresponding author on reasonable request.
